# Effect of Dexmedetomidine on Oxygen and Intrapulmonary Shunt (Qs/Qt) During One-Lung Ventilation in Pediatric Surgery: A Randomized Controlled Trial

**DOI:** 10.7759/cureus.56693

**Published:** 2024-03-22

**Authors:** Ayham Khddam, Faten Rostom, Mohammad Y. Hajeer

**Affiliations:** 1 Department of Anesthesia and Resuscitation, Children's Hospital, Damascus University, Damascus, SYR; 2 Department of Anesthesia, Faculty of Medicine, Damascus University, Damascus, SYR; 3 Department of Orthodontics, Faculty of Dentistry, Damascus University, Damascus, SYR

**Keywords:** qs/qt, pulmonary vasoconstriction for hypoventilation, one-lung ventilation, pulmonary shunt, dexmedetomidine

## Abstract

Background

One-lung ventilation (OLV) is a common ventilation technique used during thoracic surgery. It can cause serious complications in children, and hypoxic pulmonary vasoconstriction (HPV) is a protective mechanism against the resulting hypoxia. Dexmedetomidine does not affect HPV, so we will investigate its impact on the partial pressure of oxygen in arterial blood (PaO2) and pulmonary shunt fraction (Qs/Qt).

Methods

Children who underwent OLV were divided into two equal groups. The Dex group received 0.4 μg/kg/h of dexmedetomidine intravenously. The placebo group received normal saline. Two blood samples were taken to analyze arterial and central venous blood gasses during four time periods: T1, 10 minutes after anesthesia; T2, 10 minutes after OLV; T3, 60 minutes after OLV; and T4, 20 minutes after the end of OLV. Heart rate, mean arterial pressure (MAP), PaO2, Qs/Qt, and peak inspiratory pressure (PIP) values were recorded at these time points.

Results

Regarding heart rate, the Dex group remained relatively stable, whereas the placebo group showed a slight increase in T3 and T4. Concerning MAP, the Dex group had a reduction at T1 compared with the placebo group and remained similar for other points. PaO2 decreased with OLV. However, the Dex group consistently maintained higher PaO2 values than the placebo, especially in T3 and T4. Concerning Qs/Qt, the Dex group maintained lower time values than the placebo group at OLV. Regarding PIP, the Dex group had significantly lower T2 and T3 than the placebo group.

Conclusion

Administration of dexmedetomidine in children with OLV improves PaO2 and reduces pulmonary shunt fraction (Qs/Qt), thereby improving oxygen transport. It reduces the maximum PIP values, thereby reducing pressure-related complications.

## Introduction

Hypoxia during one-lung ventilation (OLV) is an important concern throughout anesthesia in thoracic surgery. OLV means mechanical separation of the two lungs with independent ventilation of one lung from the other [1] to provide a suitable space for the surgeon’s work and to protect the healthy lung from bleeding or edema caused by lung damage [2]. However, OLV can lead to a mismatch in the ventilation/perfusion (V/Q) ratio, resulting in increased pulmonary shunt fraction (Qs/Qt) within the lung as well as hypoxemia [3]. Hypoxic pulmonary vasoconstriction (HPV) is the most common and important protective mechanism against hypoxia, based on whether blood flow is transmitted from the unventilated lung to the ventilated areas to maintain adequate arterial oxygenation. HPV is affected by various factors, including changes in pulmonary pressure, alkalosis, vasodilators, and inhaled anesthetic gasses, which have the greatest effect [3]. Therefore, the lack of inhibitory effects on HPV is of great importance in anesthetizing those undergoing OLV [4,5]. It is a defense mechanism against hypoxia that reduces pulmonary stenosis [6,7].

Regarding pulmonary shunt fraction (Qs/Qt; Qs: blood flow through the shunt, Qt: total blood flow), there are some similarities between the dead space (Vd) and tidal volume (Vt) ratios and the shunt fraction Qs/Qt. Although both are involved in gas exchange (and, to some extent, related), they measure different things [8]. As blood flows through the lung, some of the blood passes through the well-ventilated alveoli and becomes fully saturated; some passes through the poorly ventilated alveoli and is only partially saturated; and some bypass the alveoli entirely. The resulting arterial oxygen content is the sum of all these compartments [9]. Dexmedetomidine is a highly selective α2-adrenergic receptor agonist. It is a derivative of clonidine, but it is eight times more potent and has no respiratory depressant effects, making it a valuable adjunct in the clinical setting [10]. It is widely used as an anesthetic adjuvant [10]. When administered at 0.3 mcg/kg/day, dexmedetomidine activates G protein and inhibits norepinephrine release in the brain. At higher doses or when administered intravenously, it stimulates peripheral α2 receptors [11]. It also has sedative, analgesic, anti-inflammatory, and organ-protective properties. It reduces sympathetic tone, heart rate (HR), blood pressure, and myocardial oxygen consumption. It also has a biphasic effect on the intrapulmonary shunt, with the shunt increasing at higher doses [12]. It is effective for postoperative pain management by reducing postoperative morphine requirements without increasing the incidence of adverse effects [13]. Adrenal alpha blockade eliminated pulmonary arrhythmias that responded to nonadrenal but not to hypoxia [14,15]. Blockade of the sympathetic nervous system does not relieve hypoxic pulmonary vascular disease. This indicates that the vasodilatory effects of dexmedetomidine increase blood flow to the abdominal regions [16]. Our study was designed as a two-group controlled clinical trial using propofol, known to have no effects on HPV, to maintain anesthesia [17-19]. Our study hypothesis predicted that dexmedetomidine would improve oxygenation and reduce pulmonary shunting during OLV in children.

## Materials and methods

Study design and settings

This study was a prospective, one-center, parallel-group, double-blind, randomized controlled trial with an allocation ratio of 1:1. This trial was started after being approved by the Scientific Research and Graduate Studies Council (Approval Number: 1810). The trial was conducted at the Children’s Hospital of the University of Damascus between March 1, 2021, and June 1, 2023. The protocol was registered at the ClinicalTrials.org registry (UDMS-Anesth-01-2021, NCT04932746). After written informed consent was obtained from the parents or legal guardians the day before surgery, 164 children were randomly assigned to receive dexmedetomidine (Dex group) or normal saline (control group) during anesthesia. Also, written informed consent was obtained from the patient’s parents/legal guardians to publish any accompanying images.

Patient recruitment and follow-up

**Figure 1:**
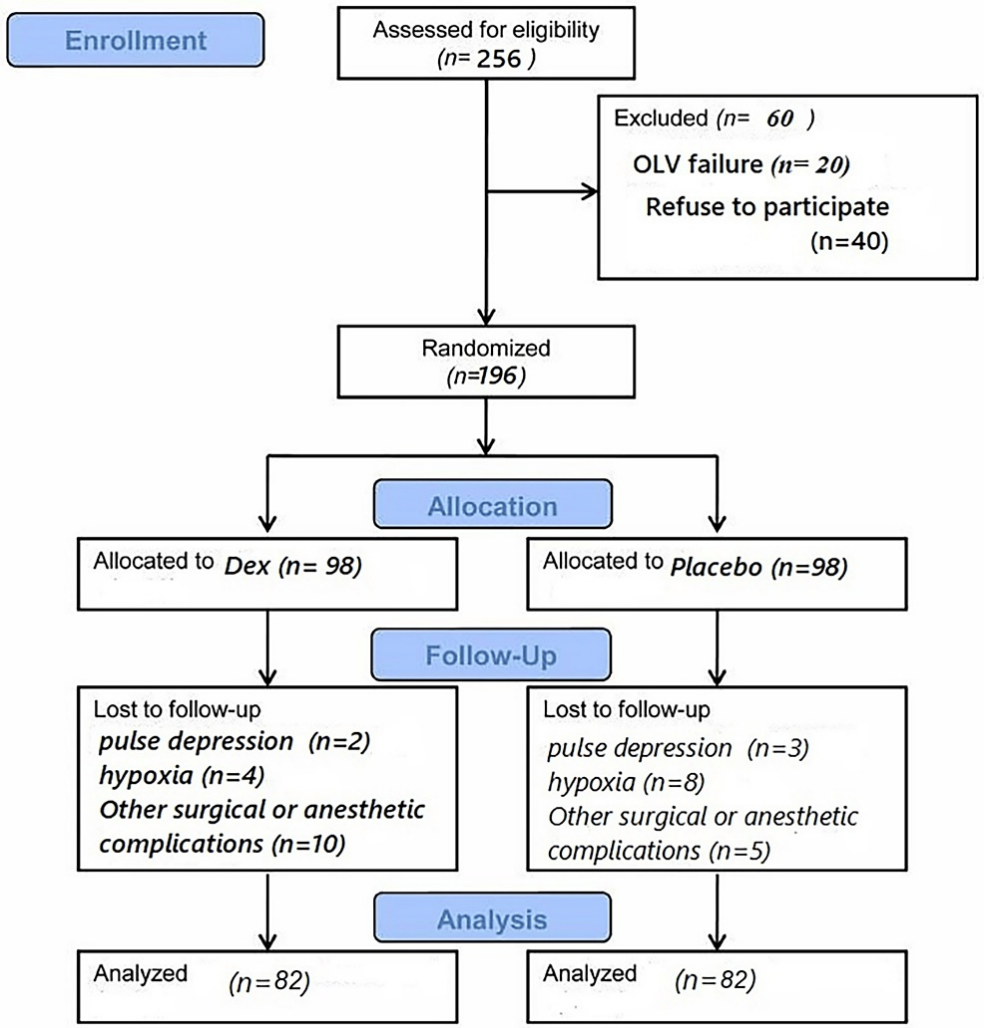
Flow diagram of patient recruitment, follow-up, and entry into data analysis OLV: one-lung ventilation.

Because no previous data were available, all patients who met the inclusion criteria and provided informed consent were included. Approximately 256 chest surgeries are performed annually in children. During the study period, 216 operations were performed using the OLV technique. All of them were deemed suitable for the study. However, 20 patients had OLV failures during surgery, and 40 refused to participate in this trial. Therefore, 196 patients were randomly assigned to the two groups. Figure 1 shows a flow chart of the patient recruitment and entry into the study.

Inclusion and exclusion criteria

The study included children with American Society of Anesthesiologists (ASA) grades I and II and children undergoing thoracic surgery with OLV from two to 12 years of age. Both genders were included in the current study. This study did not exclude practically any child who would undergo OLV, even in the presence of heart, kidney, or liver disease, provided that the degree of the disease did not reach ASA grade III. Among the exclusion criteria, the study excluded premature infants due to lung immaturity and insufficient surfactant formation. Patients with cystic fibrosis in whom surfactant and lung immaturity were not permitted to undergo OLV had no contraindications for the use of dexmedetomidine in children, except in those who showed signs of allergy to the drug. This study did not rule out practically any child who would undergo OLV. During the operating surgery, the child was excluded from the study if hypo-saturation (oxygen saturation < 90%) occurred and did not respond to maneuvers and anesthetic techniques, such as increasing the percentage of oxygen in the air being delivered to a patient (fraction of inspired oxygen, FiO2): positive-end-expiratory pressure (PEEP), tidal volume (Vt), flow, and peak inspiratory pressure (PIP). The lungs were then periodically ventilated with positive pressure, and the OLV technique was switched off if there was a drop-in heart rate of 60 beats/min that did not respond to atropine or an increased dose of cardiac tonics (dopamine). At this time, the infusion of dexmedetomidine was stopped, and it was assumed there was no very slow pulse. If it did, the primary cardiac lesion was likely the cause.

The interventional and control groups

The children who underwent OLV under general anesthesia were divided into two groups (arms): the first was administered intravenous dexmedetomidine (0.4 µg/kg/h), and the second was administered normal saline. All children were dehydrated with 2 mL/kg of normal saline before the induction of anesthesia, and routine monitoring was performed for all patients, including noninvasive blood pressure (NIBP), oxygen saturation, ECG, temperature, end-tidal carbon dioxide (EtCO2), invasive arterial blood pressure, and bispectral index (BIS). Direct anesthesia included fentanyl (1 μg/kg) and intravenous propofol (1 mg/kg) until BIS values were 50 or titrated sevoflurane to achieve a BIS of 40-60, followed by atracurium 0.5 mg/kg to facilitate endotracheal intubation. Immediately after anesthesia, dexmedetomidine was injected into the first group, and saline solution was injected into the second group. Dexmedetomidine was administered intravenously at 1 mcg/kg/hour over 10 minutes and then the infusion was continued based on the child’s weight to maintain the dexmedetomidine infusion at 0.4 mcg/kg/hour. The injection was stopped before the skin closed. The placebo group was administered the same amount of saline instead of dexmedetomidine using the same protocol. A double-lumen tube was used to isolate the lung, and the correct position of the tube was checked using a stethoscope before and after placing the child in the lateral position. For children with small-gauge isolation tubes that were not available, contralateral lung bronchial intubation was performed. A surgical maneuver after opening the chest and using retractors ensured complete isolation of the lung. A multipronged central venous catheter was placed through the right internal jugular vein on the surgical side to monitor central venous pressure and draw blood samples. Arterial blood gas (ABG) samples were obtained at four time intervals: T1: 10 minutes immediately after anesthesia and before OLV (here, we obtain the normal pulmonary arterial value); T2: 10 minutes after the start of lung isolation and OLV (before the effect of HPV reaches "15 minutes"); T3: 60 minutes after the OLV procedure to calculate the anatomical shunt value and the effect of and compare the changes between the two groups; T4: 20 minutes after the end of OLV and complete bilateral ventilation (two-lung ventilation, TLV) return. Each time, two arterial samples were taken from the radial artery or the aorta, and the second sample was taken from the pulmonary artery or the right atrium through the central catheter.

**Figure 2:**
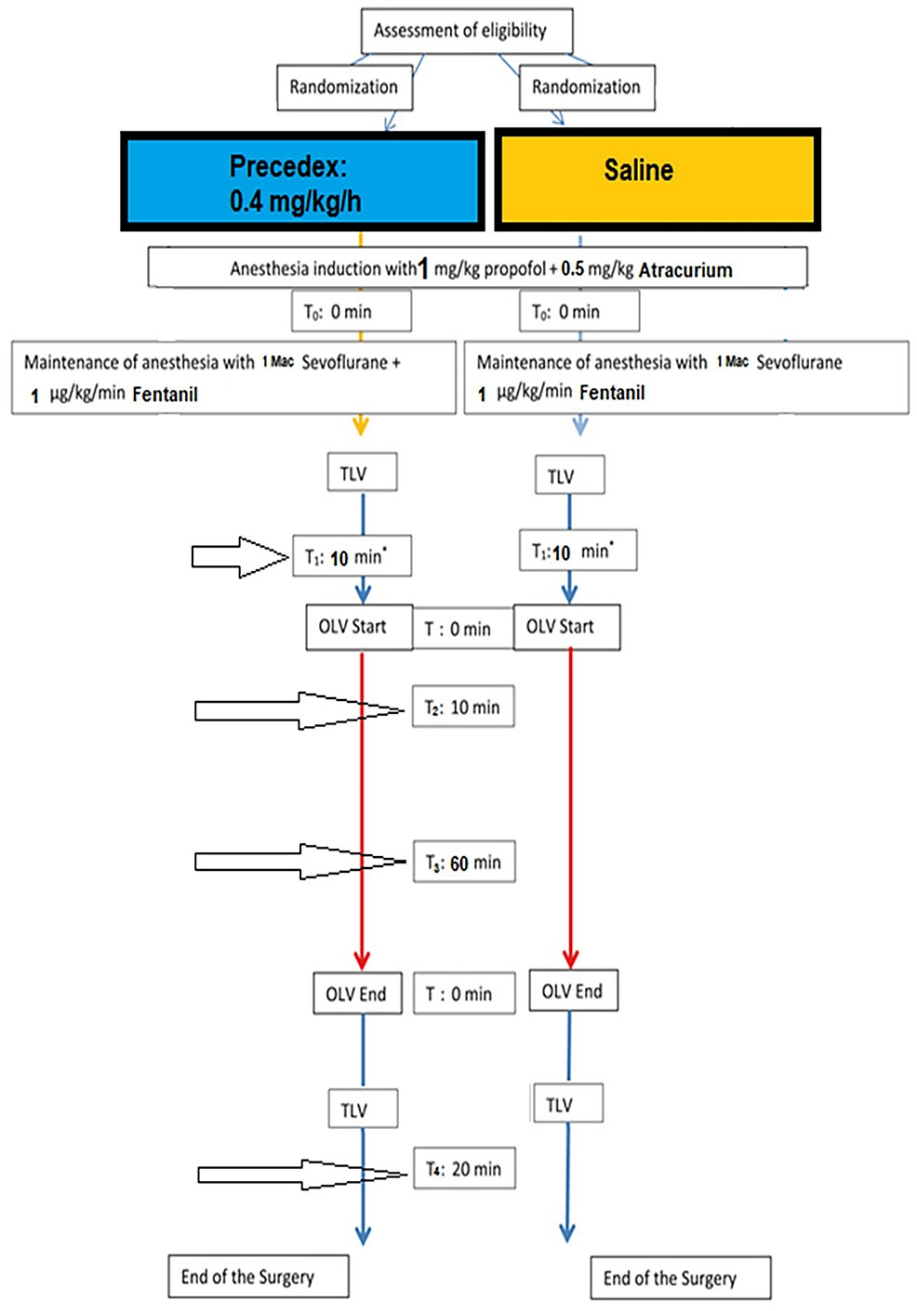
The stages of the experiment and the times for taking blood samples TLV: two-lung ventilation; OLV: one-lung ventilation.

Figure 2 shows the stages of the experiment and the times for taking blood samples for ABG analysis. During surgery, we used a 40% O2-air mixture before and after OLV and an 80% O2-air mixture during OLV. Sometimes, we needed to administer 100% oxygen for a limited time, not more than 10 minutes. Pressure-controlled ventilation was used throughout the surgery. The inducing pressure for ventilation is adjusted to provide a circulating volume that maintains end-inspiratory CO2 values of 30-35 mmHg. The pressure is then reset during the OLV. A 4-cm hydrophilic PEEP is applied throughout mechanical ventilation for all patients. Values for mechanical ventilation in OLV were set according to the child’s age and weight based on pediatric rates, rules, and schedules of Vt circulating volume, respiratory rate (RR), I:E ratio, and FiO2. The respiratory rate was adjusted to maintain CO2 (EtCO2) at approximately 35-40 mmHg. OLV was initiated after opening the pleura.

Outcome measures

The primary outcome measure is the evaluation of changes in partial pressure of oxygen in arterial blood (PaO2) levels and shunt fraction Qs/Qt during surgery using the following equation: Qs/Qt = (CcO2-CaO2)/(CcO2-CvO2), where CcO2 is the content of oxygen in pulmonary capillary blood, CaO2 is the arterial oxygen content, and CvO2 is the mixed venous oxygen content [20,21].

Randomization and blinding

One of the academic staff members not involved in this research project randomly assigned the patients into the two groups. All injections were prepared randomly by a doctor who did not participate in the study, placed in unmarked infusion pumps, and given to an anesthesiologist (more than 10 years of experience in pediatric anesthesia) without knowledge of the infusion content. The allocation concealment was performed using the sealed envelope technique. Both patients and anesthesiologists were blinded to the study drug (dexmedetomidine or placebo) by infusion of the solution. Anesthesiologists, graduate students, patients, and their families could not know to which group their child belonged. Depending on the volume in ml, mask the medical team from the substance of the drug. Infusion syringes were supplied with 0.25 mcg/mL (dexmedetomidine or placebo) at 80 mcg/20 mL, 200 mcg/50 mL, or 400 mcg/100 mL.

Statistical analysis

The null hypothesis stated no difference between the two groups in the oxygen and pulmonary shunt transportation methods. The alternative hypothesis stated that a significant difference existed between the two groups. Statistical tests were conducted to measure the extent of differences and variability between the samples. An independent-sample t-test was performed. The data were analyzed using IBM SPSS version 26 (IBM Corp., Armonk, NY). The value of p < 0.05 was considered statistically significant.

## Results

Sample characteristics

The study included 256 children after excluding 40 children because of refusal to participate, 20 children because of the inability of good bronchial isolation, five children because of unresponsive pulse depression (two in the Dex group and three in the placebo group), 12 children because of unresponsive hypoxia (four in the Dex group and eight in the placebo group), and 15 children because of the occurrence of important surgical or anesthetic complications such as anaphylactic shock resulting from an explosion hydatid cyst (10 in the Dex group and five in the placebo group). The study was completed by 164 children (82 children per group). There were no significant differences in sex between the two groups (p > 0.05). Table 1 shows the distribution of children of the study patients regarding gender.

**Table 1:** Distribution of children in the two groups in terms of gender

Groups			Dex	Placebo	All children	P
Gender	Male	N	39	38	77	0.710
		Percent (%)	47.56%	46.34%	46.95%	
	Female	N	43	44	87	0.806
		Percent (%)	52.44%	53.66%	53.05%	
	All		82	82	164	

Patient details and pulmonary function tests were similar between the two groups. The baseline (BL) mean values were comparable between the groups. There were no significant differences in demographic and baseline variables between the two groups (p > 0.05) regarding age, body mass index (BMI), operational time, crystalloid and colloidal infusion, arterial blood gas (ABG), including pH BL, PaO2 BL, and PaCo2 BL, blood hemoglobin (Hg BL), heart rate (HR BL), mean arterial pressure (MAP BL), and pulmonary function tests (forced expiratory volume in one second (FEV1)/forced vital capacity (FVC)/% BL). Despite the difficulty of taking these measurements in children and the difficulty of adopting them, the differences between the two groups were not significant (p > 0.05; Table 2).

**Table 2:** Demographic and baseline data for all patients in the two groups with the p-value of significant testing BL: baseline; PaO2: partial pressure of oxygen in arterial blood; PaCo2: partial pressure of carbon dioxide; Hg: hemoglobin; HR: heart rate; MAP: mean arterial pressure; FEV1: forced expiratory volume in one second; FVC: forced vital capacity.

Groups		Mean	Standard deviation	P-value
Age (years)	Dex	7.6099	2.26709	0.746
	Placebo	7.4745	2.44947	
Body mass index (kg/m2)	Dex	6.2102	0.57671	0.399
	Placebo	6.1253	0.55811	
Operational time (min)	Dex	112.06	20.600	0.233
	Placebo	116.95	25.305	
Crystalloid (l/kg)	Dex	9.4934	3.12926	0.202
	Placebo	8.8100	2.89581	
Colloid (ml/kg)	Dex	7.2588	2.33644	0.952
	Placebo	7.2835	2.26172	
Urine output (ml/kg）	Dex	2.7879	0.81606	0.957
	Placebo	2.7950	0.64928	
PH BL	Dex	7.3938	0.02433	0.662
	Placebo	7.3956	0.02409	
PaO_2_ BL (mmHg)	Dex	81.59	5.514	0.197
	Placebo	82.92	6.046	
PaCo2 BL (mmHg)	Dex	37.09	1.205	0.477
	Placebo	37.27	1.504	
Hg BL (mg/dL)	Dex	11.8531	1.21237	0.525
	Placebo	11.7188	1.17458	
HR BL (beats/min)	Dex	112.74	8.072	0.664
	Placebo	113.45	9.107	
MAP BL (mmHg)	Dex	74.14	9.96	0.343
	Placebo	74.77	10.77	
FEV1/FVC/% BL	Dex	83.2678	3.22285	0.296
	Placebo	82.6772	3.14040	

Main findings

Table 3 shows the measured values during OLV comparing the Dex and placebo groups undergoing OLV in pediatric thoracic surgery. This table shows the measurements taken at four different time points (T1: 10 minutes after anesthesia and total ventilation; T2: 10 minutes after OLV; T3: 60 minutes after OLV; T4: 20 minutes after resuming bilateral ventilation TLV).

**Table 3:** Descriptive statistics of the collected variables in the two groups across the different assessment times TLV: two-lung ventilation; OLV: one-lung ventilation; PaO2: partial pressure of oxygen in arterial blood; HR: heart rate; MAP: mean arterial pressure; PEEP: positive-end-expiratory pressure.

Groups		T1 TLV 10			T2 OLV 10			T3 OLV 60			T4 Re TLV		
		Mean	SD	P-value	Mean	SD	P-value	Mean	SD	P-value	Mean	SD	P-value
HR (beats/min)	Dex	110.74	8.072	0.664	114.94	17.151	0.172	112.49	18.823	0.025	113.95	11.739	0.042
	Placebo	111.45	10.107		119.53	20.515		120.03	18.678		118.87	15.165	
MAP (mmHg)	Dex	72.23	9.335	0.042	73.06	11.038	0.868	74.34	9.968	0.343	79.46	9.424	0.792
	Placebo	76.78	9.635		74.70	13.160		76.17	11.777		79.93	10.672	
PaO_2_ (mmHg)	Dex	352.52	50.454	0.893	190.22	46.446	0.285	232.10	38.783	0.000	354.19	35.631	0.036
	Placebo	351.34	48.293		181.63	44.067		195.60	52.436		340.92	35.181	
Qs/Qt	Dex	9.603	2.0758	0.648	23.6469	2.58800	0.000	21.759	3.3397	0.000	9.613	0.6457	0.000
	Placebo	9.759	1.7801		30.2000	2.73287		27.566	1.3031		11.619	1.2024	
P peak (cmH_2_O)	Dex	14.68	1.105	0.472	17.68	3.630	0.034	15.45	3.246	0.000	17.39	4.693	0.874
	Placebo	14.81	0.990		19.47	5.626		20.66	5.361		17.53	5.324	
PEEP (cmH_2_O)	Dex	2.67	0.977	0.459	3.09	1.330	0.851	3.09	1.330	0.851	3.06	1.424	0.661
	Placebo	2.55	0.925		3.14	1.479		3.14	1.479		3.17	1.386	

Heart Rate (HR)

**Figure 3:**
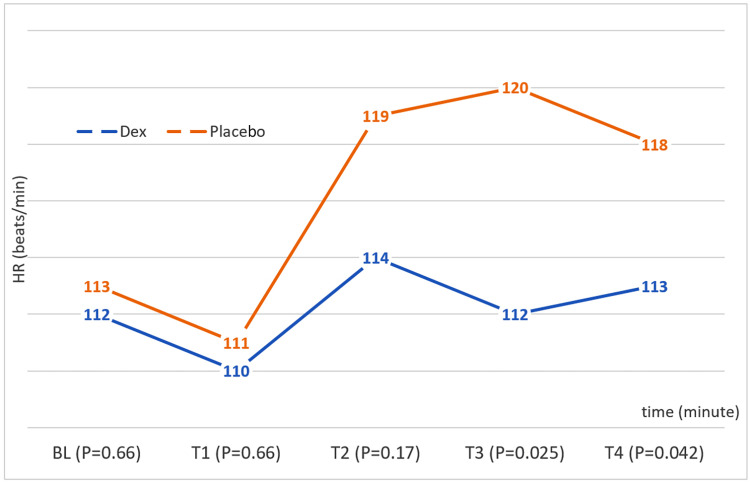
HR (beats/min) changes in children during surgery according to the time (minute) and group BL: baseline; HR: heart rate.

In the Dex group, HR remained relatively stable throughout, whereas the placebo group showed a slight increase during OLV. Significant differences were observed at T3 and T4 between the Dex and placebo groups. There were no differences between the first and second periods; statistically significant differences appeared 60 minutes after lung isolation and after the isolation ended (T3: Dex = 112.49 ± 18.82 beats/min, placebo = 120.03 ± 18.67 beats/min; T4: Dex = 113.95 ± 11.73 beats/min, p = 0.025, placebo = 118.87 ± 15.17 beats/min, p = 0.045). Figure 3 shows the HR (beats/minute) and changes in children during surgery according to the time and group.

Mean Arterial Pressure (MAP)

The Dex group had a statistically significant decrease in MAP at T1 compared with the placebo group. MAP values remained similar between the groups for the remaining time points.

**Figure 4:**
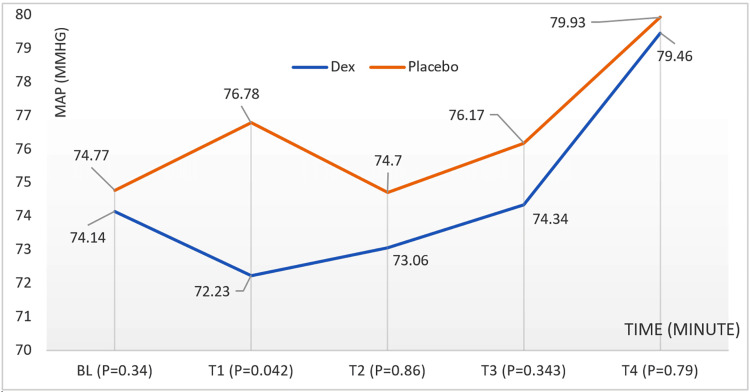
MAP (mmHg) changes in children during surgery according to the time (minute) and group BL: baseline; MAP: mean arterial pressure.

Fifteen minutes after the anesthesia and before the isolation procedure, no differences occurred between the two groups except for the MAP (mmHg) drop in the Dex group, which did not happen with the placebo group, by no more than 10% from BL (Dex BL = 74.14 ± 9.96), with a statistically significant difference (T1: Dex = 72.23 ± 9.34 mmHg, placebo = 76.78 ± 9.64 mmHg, p = 0.042). After that, no differences were observed in pressure between the two groups. Figure 4 shows children's MAP changes during surgery according to the time (minutes) and group.

Partial Pressure of Oxygen in Arterial Blood (PaO2)

**Figure 5:**
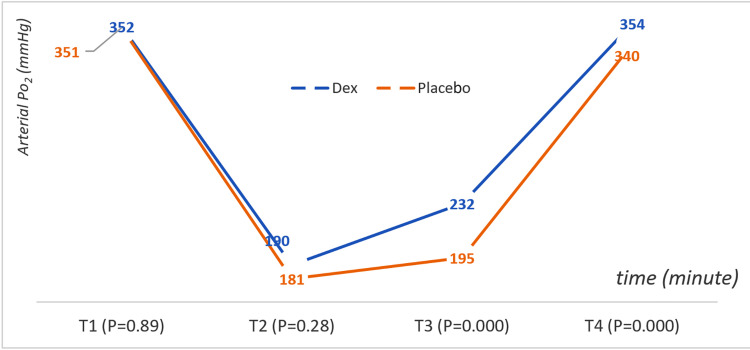
Arterial PaO2 (mmHg) changes in children during surgery according to the time (minute) and group PaO2: partial pressure of oxygen.

The Dex group maintained consistently higher PaO2 values than the placebo group, especially at T3 and T4. This indicated improved oxygenation in the Dex group. The PaO2 (mmHg) decreased with OLV. There was no difference before the OLV procedure and even 10 minutes after the OLV, but one hour after the OLV, there were very significant statistical differences (T3: Dex = 232.10 ± 38.78 mmHg and placebo = 195.60 ± 52.44 mmHg, with p < 0.001). At the end of surgery and return to double ventilation, there were significant statistical differences between the two groups (T4: Dex = 354.19 ± 35.64 mmHg and placebo = 340.92 ± 35.18 mmHg, with a value of p = 0.036). Figure 5 shows arterial PaO2 (mmHg) changes in children during surgery according to the time (minutes) and groups.

Pulmonary Shunt Fraction (Qs/Qt)

**Figure 6:**
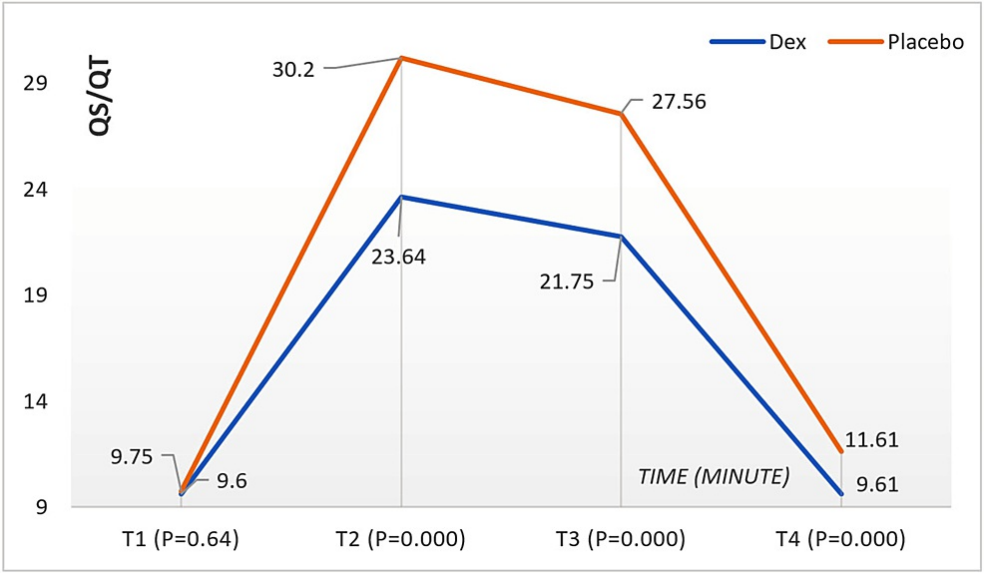
Qs/Qt changes in children during surgery according to the time (minute) and group

The Dex group maintained significantly lower Qs/Qt values at all time points except T1 than the placebo group. This result indicated reduced pulmonary shunt and enhanced oxygen transport in the Dex group. The value of (Qs/Qt) increased after the isolation OLV procedure, and very significant statistical differences appeared between the two groups after 10 minutes and 60 minutes of starting the OLV (T2: Dex = 23.65 ± 2.59, placebo = 30.20 ± 2.73, p < 0.001; T3: Dex = 21.76 ± 3.34, placebo = 27.57 ± 1.30, p < 0.001) and after the end of OLV and isolation and return to double ventilation of the lungs, the statistical differences remained very significant between the two groups (T4: Dex = 9.61 ± 0.65, placebo = 11.62 ± 1.20, p < 0.001). Figure 6 shows Qs/Qt changes in children during surgery according to the time (minutes) and groups.

Positive End-Expiratory Pressure (PEEP) and Peak Inspiratory Pressure (P Peak)

**Figure 7:**
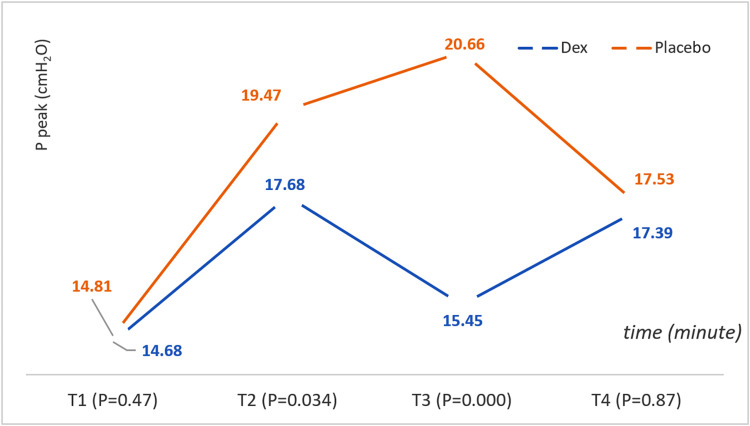
P peak (cmH2O) changes in children during surgery according to the time (minute) and group P peak: peak inspiratory pressure.

Similar values were observed between both groups throughout the study. The P peak refers to the peak inspiratory pressure, the highest airway pressure reached during inhalation in mechanical ventilation. The Dex group significantly decreased the P peak at T2 compared with the placebo group. This indicates a potentially lower risk of pressure-related complications in the Dex group. Concerning mechanical ventilation, there were no significant statistical differences between the two groups in terms of PEEP (cmH2O), but in terms of P peak (cmH2O), we saw significant statistical differences after OLV lung isolation and throughout the OLV period. The group Dex values were significantly lower (T2: Dex = 17.68 ± 3.63 cmH2O, placebo = 19.47 ± 5.63 cmH2O, p = 0.034; T3: Dex = 15.45 ± 3.25 cmH2O, placebo = 20.66 ± 5.36 cmH2O, p < 0.001). Figure 7 shows P peak (cmH2O) changes in children during surgery according to the time (minute) and group.

From the previous table, the dexmedetomidine infusion during OLV in pediatric thoracic surgery offered the following benefits: (1) improved oxygenation: higher PaO2 values in the Dex group indicated better oxygen delivery to tissues, crucial for maintaining vital organ function during surgery. (2) Reduced pulmonary shunting: lower Qs/Qt values in the Dex group indicated enhanced blood flow through functional lung units and reduced bypassing through non-ventilated areas, thereby improving the overall efficiency of oxygen transport. (3) Stable hemodynamics: the group receiving dexmedetomidine did not show significant alterations in MAP or HR, indicating that dexmedetomidine does not hurt blood pressure or heart rate. This suggests good tolerability of dexmedetomidine at the recommended dosage. (4) Reduced pressure-related complications: the reduced P peak in the Dex group may indicate less strain on the respiratory system, airways, and chest during ventilation, which is beneficial for minimizing trauma and promoting faster recovery.

## Discussion

To our knowledge, this is the first study conducted on children in the Middle East and West Asia. We have used dexmedetomidine with sevoflurane as an inhalation anesthetic in thoracic surgeries in children in whom single lung ventilation (OLV) is performed. We did not find similar studies in children; only one similar study was conducted in adults [22]. Wang et al. administered a loading dose of 0.75 µg/kg over 10 minutes, followed by a maintenance dose of 0.3 µg/kg/hour, and made measurements over three periods to determine the effect of dexmedetomidine [22]. The results showed that his shunt improved, as the Qs/Qt ratio was reduced with a statistically significant difference, but his oxygen transport (PaO2) was not significantly affected. The effect of dexmedetomidine on the anesthetic agent sevoflurane was also studied, and it was found that the anesthetic requirements for sevoflurane were reduced in the dexmedetomidine group compared with the placebo group.

Children's special anatomy and physiology include small ventricles with low systolic mass and poor ventricular compliance [23]. With decreased vascular resistance surrounding systemic vascular resistance (SVR) [24], the higher cardiac output in adults depends on HR [25,26] and the fact that the pulmonary vessels maintain an important reserve blood volume that they circulate if they contract to compensate for the loss of volume [27]. In addition, the parasympathetic nervous system is dominant [28]. This explains why HR is affected compared with changes in mean blood pressure, which is less affected and slower to respond [27,29].

This explains the decrease in HR in our experiment in response to the administration of dexmedetomidine, which acts on central and peripheral α2 receptors and prevents sympathetic responses, and this appeared especially in statistical differences with the progression of time T3 and T4 after 60 minutes of lung isolation and after the isolation ended. No significant decrease in MAP was observed between the two groups except at T1, 10 minutes after the dexmedetomidine infusion and before the isolation procedure, which did not occur with the placebo group, at a rate not exceeding 10% of Dex BL, with a statistically significant difference. Its decrease was initially explained as a response to dexmedetomidine, but the initiation of surgery, isolation, and the occurrence of HPV vasoconstriction of the pulmonary vessels quickly compensated for it.

The excessive dose of dexmedetomidine can reduce blood pressure and HR, which is not conducive to organ perfusion and patient safety, in the experiment, for this dose of 0.4 µg/kg/hour, during the OLV process. During thoracic surgery, when the patient is lying on his/her side, OLV can easily cause breathing/blood flow disturbance, increased chest pressure on the ventilated side, and thus decreased return blood flow. Preoperative and surgical administration of dexmedetomidine can control hemodynamic stability and have a protective effect on the cardiovascular system in the intraoperative period [30]. This is consistent with the findings in our study of chest surgery using the OLV technique, where we observed stabilization of the blood circulatory system, cardiac rhythm, HR, and MAP in the group that received dexmedetomidine during surgery.

The Dex group maintained consistently higher PaO2 values than the placebo group, especially at T3 and T4. This indicates improved oxygenation in the Dex group. Respiratory anatomical and physiological considerations in children explain the improvement of oxygen transport with the application of dexmedetomidine [31], as each terminal bronchiole opens into a single alveolus and initially forms uveal clusters [32], rather than fully developed alveolar clusters [33]. The alveoli are thick-walled and comprise only 10% of all adults. These complexes continue to grow until six to eight years to adult resemblance [34]. The cartilaginous ribs are aligned horizontally so that they do not have a “bucket handle” action for the chest, as in adults [35]. The intercostal muscles are poorly developed, with a lower proportion of type 1 muscle fibers, and fatigue occurs more easily [11,32]. The diaphragm is more horizontal, which reduces its mechanical advantage. Ventilation depends mainly on the diaphragm and the rate of its movements (breathing movements) [36]. High chest wall compliance due to cartilaginous chest and intercostal and sternal instability is common with increased respiratory work or airway obstruction [35]. Most importantly, a decreased functional residual capacity (FRC) increases the pulmonary shunt and leads to atelectasis [37,38].

Dillon et al., in a literature review, noted four out of seven studies by Asri et al. [39], Xu et al. [40], Xia et al. [7], and Lee et al. [3] presented statistically significant improvement in oxygenation among patients receiving dexmedetomidine during OLV [41]. Therefore, co-administration of dexmedetomidine may be considered an appropriate intervention to reduce the degree of hypoxia in lung surgeries if maintenance of oxygenation and prevention of inflammatory changes in the lungs during surgery can lead to improved postoperative outcomes and reduced hospital stay [41]. This is consistent with the results of this study. Jiang et al. showed that dexmedetomidine could effectively alleviate inflammatory response and oxidative stress, reduce oxygen requirement, lower shunt and Qs/Qt ratio, and improve PaO2 and lung function during OLV with good effects at different time points [42]. These results are similar to those of our study.

The Dex group maintained significantly lower Qs/Qt values at all time points except T1 than the placebo group. The results revealed that dexmedetomidine can reduce the intrapulmonary shunt and improve the HPV-induced vasoconstriction response. It greatly enhances the state of PaO2 during pulmonary ventilation. Previous studies have shown that dexmedetomidine combined with isoflurane can improve vasoconstriction caused by decreased ventilation and body oxygenation during OLV [43], consistent with our study.

Other studies have shown that vasoconstrictive drugs, such as adrenergic receptor agonists, can enhance vasoconstriction resulting from decreased ventilation [44,45]. For example, dexmedetomidine enhances HPV hypoventilation-induced vasoconstriction by directly activating peripheral α2 receptors and thus causes pulmonary vasoconstriction during OLV. Xia et al., in 2015, showed that dexmedetomidine enhances the vasoconstrictor effect of decreased ventilation by reducing the level of oxidative stress in the body during single-lung ventilation, thus improving the oxygenation status of patients [7].

Janković et al., in 2019, mentioned that OLV carries a risk of hypoxia and hypercapnia due to intrapulmonary shunting and dead space ventilation [46]. Both can significantly impact the management of perioperative anesthesia and postoperative complications. Today’s technologies and medications can mitigate the consequences of OLV.

The key to preventing postoperative complications during thoracic surgery is to apply the correct ventilator strategy. However, preventative ventilation appears appropriate to lower the risk of lung damage and acute lung injury (ALI), although no published guidelines exist. Despite a growing awareness of the importance of preventive ventilation, many clinicians still use running volume and maximum pressure outside the recommended levels in daily practice [46].

Here, the importance of using dexmedetomidine appears to be significantly reduced in reducing the need for high pressures used during mechanical ventilation to ensure adequate oxygenation, as the P peak value recorded in our study decreased in the group of patients who received dexmedetomidine during the operation. As for the second group that did not apply dexmedetomidine, we had to increase the pressure (P peak), according to Janković et al. [46]. Bai et al. [47] and Wu et al. [48] showed that dexmedetomidine reduces the inflammatory response during lung ventilation, thereby reducing lung injury. This effect may be associated with improved intrapulmonary shunting. Regarding mechanical ventilation, there were no statistically significant differences between the two groups regarding PEEP, but in terms of P peak, we observed statistically significant differences after OLV lung isolation and throughout the OLV period. The Dex group values were significantly lower because the Dex group did not need high pressures to reach an appropriate oxygenation level compared with the placebo, and this is due to improved oxygen transfer to them.

Limitations of the current study

The high price of dexmedetomidine and its unavailability (during the crisis in Syria) were the main obstacles to the study, especially considering the absence of other supporting bodies. The study included only one medical center (University Children's Hospital) because this type of operation requires surgical and anesthesia personnel specialized in performing chest surgery in children, isolating the lungs, and anesthetizing children during single-lung ventilation.

Further research with larger populations is required to confirm these findings and establish optimal dosing strategies for dexmedetomidine in this setting. This study investigated the administration of a solitary dosage. We propose exploring alternative dosages in pediatric patients and conducting a comparative analysis of these dosages. Demographic variables, basic pediatric variables, and pulmonary function tests were similar between the two groups. The baseline was comparable between the groups. We maintained adequate airway pressure and peripheral blood oxygen saturation during OLV. All unsuccessful or incomplete OLV isolations within the inclusion criteria were excluded. There were no significant differences in PaO2 BL and Qs/Qt BL between the two groups at T0. Therefore, we considered dexmedetomidine to be the primary agent affecting the intrapulmonary shunt and HPV during OLV in our study.

## Conclusions

This study provides evidence that intraoperative dexmedetomidine infusion on a single lung in pediatric thoracic surgery has several benefits in improving the level of oxygen in the arteries and leading to better delivery of oxygen to the tissues, reducing pulmonary shunting with improved blood flow across functional lung units and reduced overflow across non-ventilated areas. Also, it produces stable hemodynamics since there were no significant changes in MAP or HR, indicating good tolerance within the recommended dose. Additionally, the intraoperative dexmedetomidine reduced the risk of complications related to pressure within the airways and chest due to the low pressures required to achieve good oxygenation. Dexmedetomidine is a promising agent for improving oxygenation, reducing pulmonary shunt, and potentially reducing the risk of complications during OLV in pediatric thoracic surgery.
